# Supplementary material for: Ribosome biogenesis in plants requires the nuclear envelope and mitochondria localized OPENER complex
Source: Nat Commun. 2025 Aug 7;16:7301. doi: 10.1038/s41467-025-62652-7 (PMC12332008; doi:10.1038/s41467-025-62652-7)
Supplement: Supplementary file 1 — Supplementary Figs. and table [file 41467_2025_62652_MOESM1_ESM.pdf]

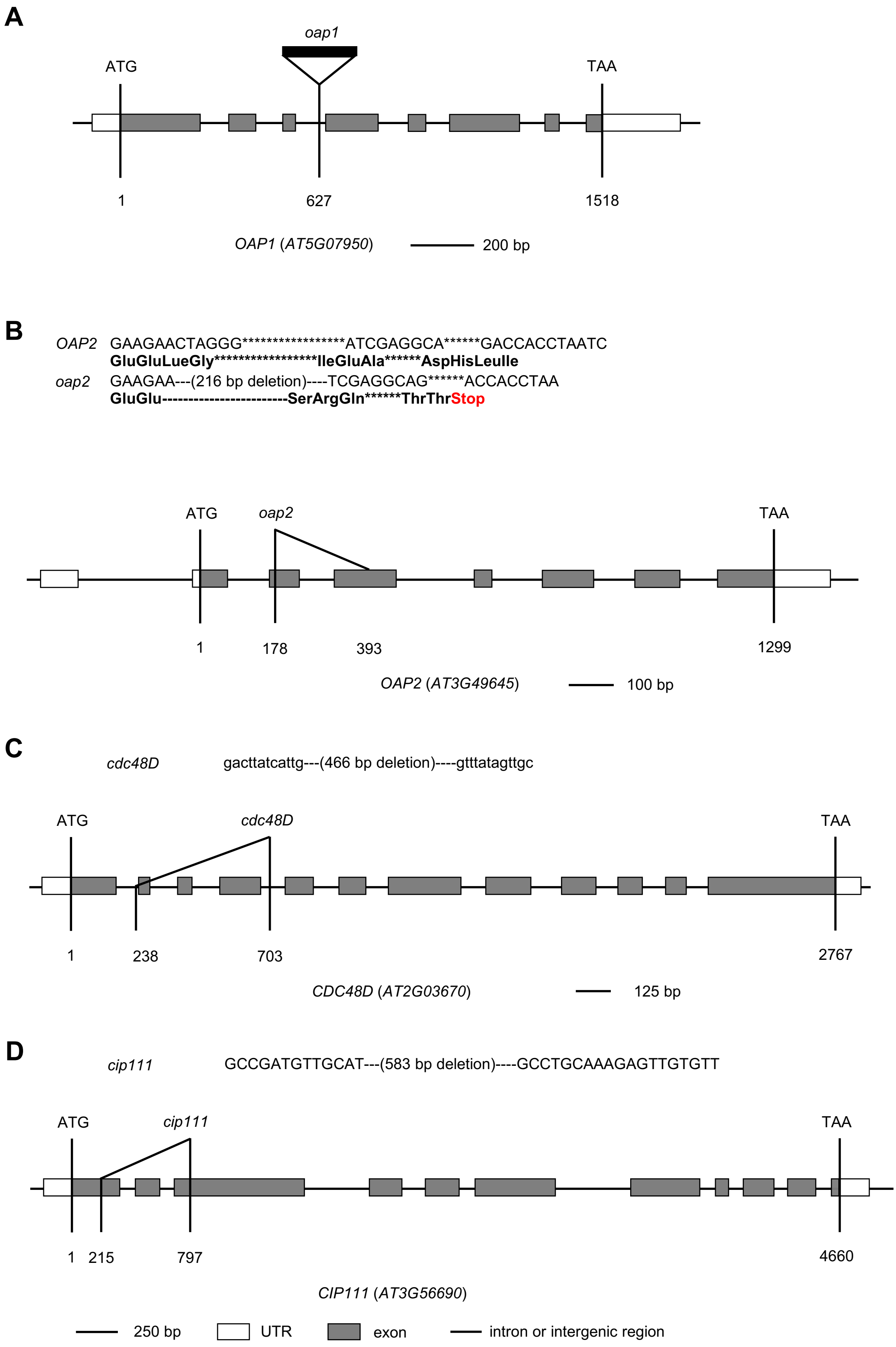

Supplementary Fig. 1. Mutants of *oap1*, *oap2*, *cdc48D* and *cip111*.
Schematic diagrams of the gene structures and mutation sites of *OAP1* **(A)**, *OAP2* **(B)**, *CDC48D* **(C)** and *CIP111* **(D)**. The white and grey boxes indicate untranslated and translated regions, respectively.

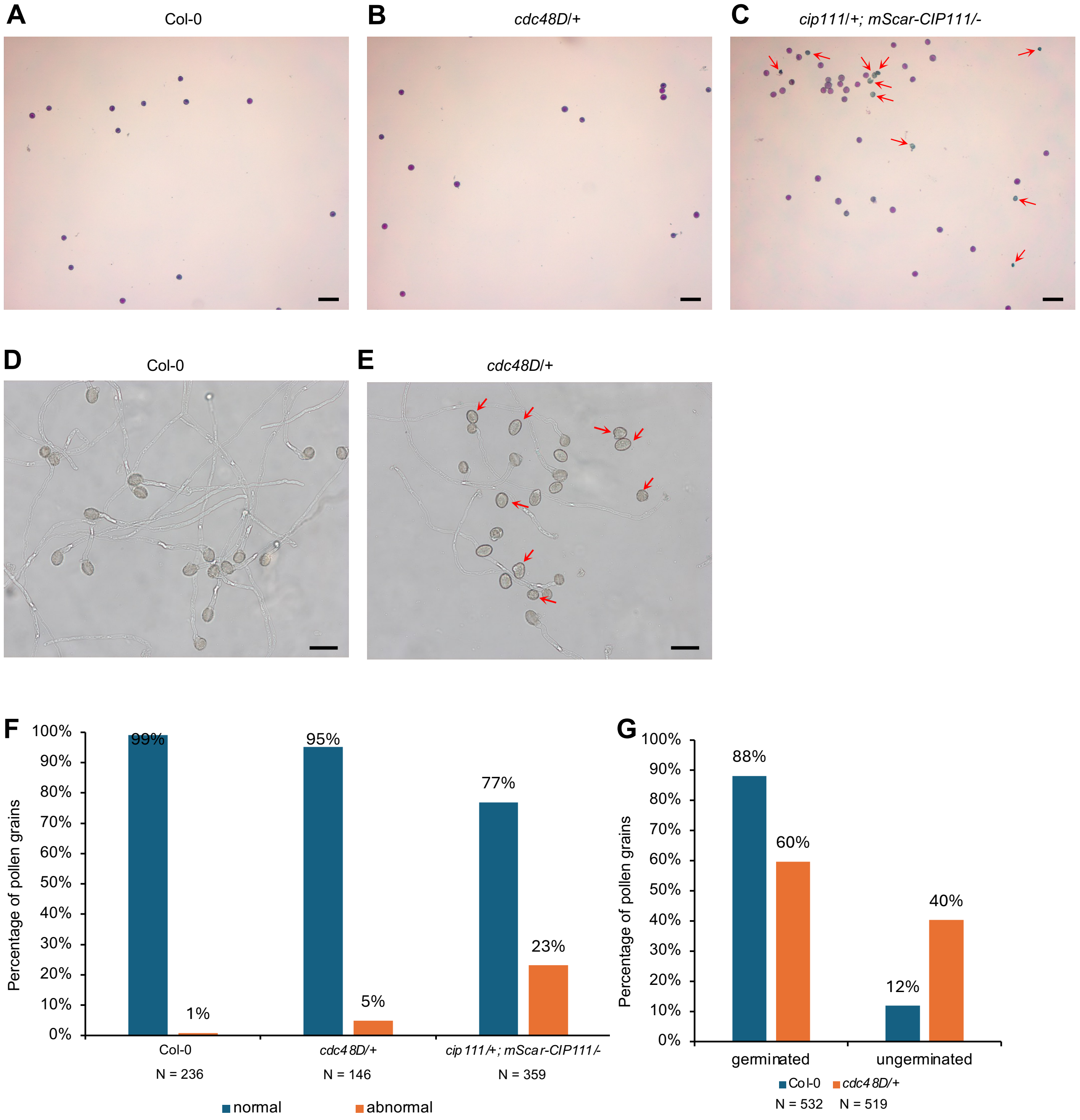

Supplementary Fig. 2. *CDC48D* and *CIP111* knockout results in male gametophyte defects.
(A) to **(C)** Alexander staining of pollen from Col-0 **(A)**, *cdc48D*/+ **(B)** and *cip111*/+; *mScar-CIP111/-* **(C)** plants. Aborted pollen grains are stained green and indicated by red arrows.
(D) and **(E)** *In vitro* germination of pollen grains from Col-0 **(D)** and *cdc48D*/+ **(E)** plants. Red arrows indicate ungerminated pollen grains.
(F) Percentage of normal and abnormal pollen grains from Col-0 (*n* = 236), *cdc48D*/+ (*n* = 146) and *cip111*/+; *mScar-CIP111/-* (*n* = 359) plants.
(G) Percentage of germinated and ungerminated pollen grains from Col-0 (*n* = 532) and *cdc48D*/+ (*n* = 519) plants.
Scale bars: **(A)** to **(C)**: 20 µm. **(D)** and **(E)**: 50 µm.

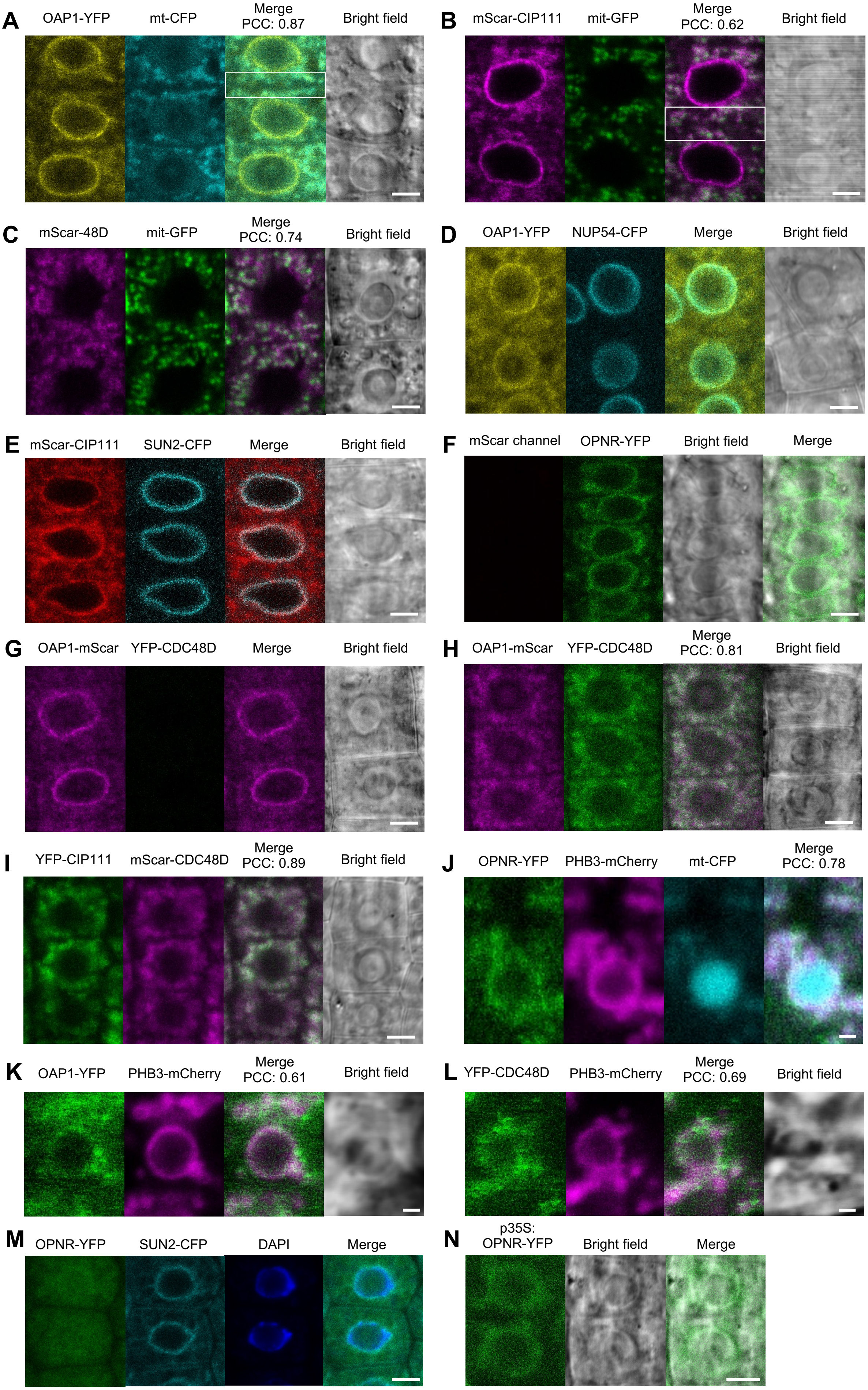
A
OAP1-YFP
mt-CFP
Merge
PCC: 0.87
Bright field
B
mScar-CIP111
mit-GFP
Merge
PCC: 0.62
Bright field
C
mScar-48D
mit-GFP
Merge
PCC: 0.74
Bright field
D
OAP1-YFP
NUP54-CFP
Merge
Bright field
E
mScar-CIP111
SUN2-CFP
Merge
Bright field
F
mScar channel
OPNR-YFP
Bright field
Merge
G
OAP1-mScar
YFP-CDC48D
Merge
Bright field
H
OAP1-mScar
YFP-CDC48D
Merge
PCC: 0.81
Bright field
I
YFP-CIP111
mScar-CDC48D
Merge
PCC: 0.89
Bright field
J
OPNR-YFP
PHB3-mCherry
mt-CFP
Merge
PCC: 0.78
K
OAP1-YFP
PHB3-mCherry
Merge
PCC: 0.61
Bright field
L
YFP-CDC48D
PHB3-mCherry
Merge
PCC: 0.69
Bright field
M
OPNR-YFP
SUN2-CFP
DAPI
Merge
N
p35S:
OPNR-YFP
Bright field
Merge

Supplementary Fig. 3. OAP1, CDC48D and CIP111 subcellular localization.
(A) to **(C)**: Confocal laser scanning microscope (CLSM) images of root tip cells from seedlings stably expressing mt-CFP and *pOAP1:OAP1-YFP* **(A)**, mit-GFP and *pCIP111:mScarlet-CIP111m* **(B)** and mit-GFP and *pCDC48D:mScarlet-CDC48D* **(C)**. mt-CFP and mit-GFP are mitochondria maker lines. The Pearson correlation coefficient (PCC) was shown for the area in the white boxes in **(A)** and **(B)**. PCC in **(C)** was shown for the whole image.

(D) CLSM images of root tip cells from seedlings stably expressing *pUBQ10:NUP54-CFP* and *pOAP1:OAP1-YFP*. NUP54 is a component of the nuclear pore complex and NUP54-CFP are marker of nuclear envelope.

(E) CLSM images of root tip cells from seedlings stably expressing *pSUN2:SUN2-CFP* and *pCIP111:mScarlet-CIP111*. SUN2 is an inner nuclear membrane protein and SUN2-CFP is a nuclear envelope marker.

(F) Images of root tip cells from seedlings only expressing OPNR-YFP in the mScar-CDC48D x OPNR-YFP F2 segregation population.

(G) and **(H)**: CLSM images of root tip cells from seedlings harboring *pRPS5A:XVE:YFP-CDC48D* and *pOAP1:OAP1-mScarlet* before **(G)** and after **(H)** *YFP-CDC48D* induction. The same settings of YFP and mScarlet channels were used for **(G)** and **(H)**. There is almost no YFP-CDC48D signals before inducing in **(G)**. PCC was shown for the colocalization between YFP-CDC48D and OAP1-mScarlet in **(H)**.

(I) CSLM images of root tip cells from seedlings stably expressing *pCIP111:YFP-CIP111* and *pCDC48D:mScarlet-CDC48D*. PCC was shown for the colocalization between YFP-CIP111 and mScarlet-CDC48D in **(H)**.

(J) to (L) CLSM images of root tip cells from seedlings stably expressing mt-CFP, PHB3-mCherry and OPNR-YFP **(J)**, PHB3-mCherry and OAP1-YFP **(K)** and PHB3-mCherry and YFP-CDC48D **(L)**. PHB3 is a mitochondria inner membrane protein and PHB3-mCherry is a mitochondria inner membrane marker. PCC was shown for the colocalization of OPNR-YFP and PHB3-mCherry in **(J)**, PHB3-mCherry and OAP1-YFP in **(K)** and PHB3-mCherry and YFP-CDC48D in **(L)**.

(M) CLSM images of 4% formaldehyde fixed and DAPI strained root tip cells from seedlings stably expressing *pSUN2:SUN2-CFP* and *pOPNR:OPNR-YFP*.

(N) Root tip cells stably expressing *p35S: OPNR-YFP*.

Scale bars: **(A)** to **(I)**, **(M)** and **(N)**: 5 µm, **(J)** to **(L)**: 1 µm.

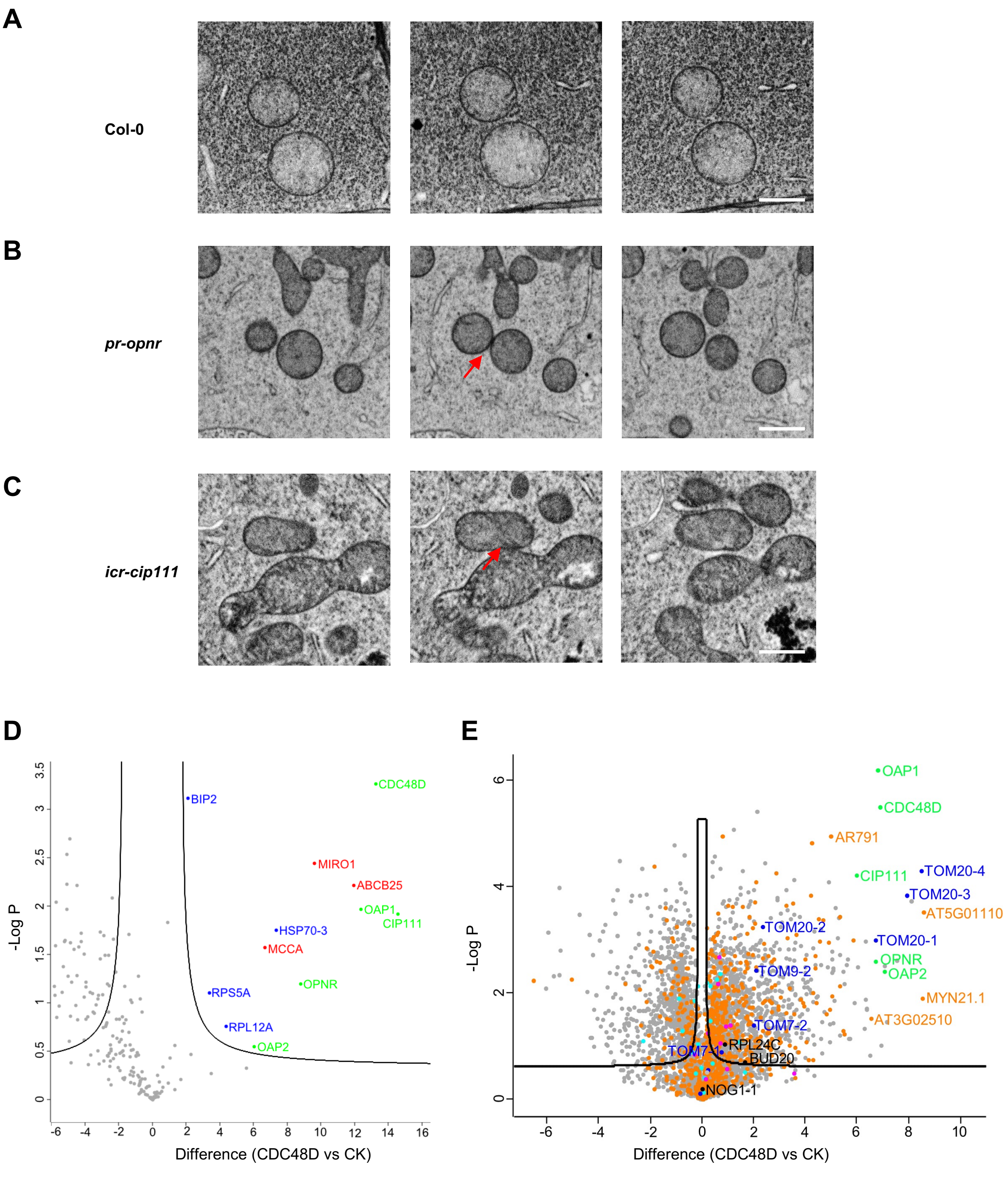

Supplementary Fig. 4. Focused ion beam scanning electron microscopy (FIB-SEM) acquired images and proteomic analyses of the samples from TurboID-YFP-CDC48D and TurboID-YFP control of the mitochondrial fractions.

(A) to **(C)** FIB-SEM serial images of root cells from Col-0 **(A)**, *pr-opnr* **(B)** and *icr-cip111* **(C)** seedlings. Different sections were presented from left to the right. Red arrows indicate contact sites between neighbouring mitochondria.

(D) GFP-Trap co-IP and mass spectrometry results with TurboID-YFP-CDC48D (CDC48D) and TurboID-YFP (CK) serving as baits. Total protein isolated from mitochondria fraction was used. Volcano plots for CDC48D vs CK with the -log10*P*-value shown on the y-axis and log2 intensity differences between the comparisons shown on the x-axis. Each dot represents a protein. Red, blue and green dots represent significantly enriched proteins in YFP-CDC48D. Components of the OPNR complex were shown in green. Mitochondrial proteins were shown in red. Cytosolic proteins were shown in blue. *P*-values were calculated using two-tailed Student's *t*-test, moderated by Benjamini–Hochberg's method. FDR = 0.1, s0 = 2. The full lists are shown in Dataset S1A.

(E) Volcano plot shows the –log10*P*-value (y axis) and log2 intensity differences (x axis) of TurboID-YFP-CDC48D (CDC48D) and TurboID-YFP (CK). Total protein isolated from mitochondria fraction was used. The intensity of each protein was normalized to the total signal of each sample. Each dot represents a protein. Green dots represent proteins of the OPNR complex. Orange, blue and purple dots represent mitochondrial proteins encoded by the nuclear genes. Cyan dots represent mitochondria genome encoding proteins. Blue dots represent subunits of the TOM complex. Purple dots represent subunits of the TIM complex. RPL24C, NOG1-1 and BUD20 are shown in black. *P*-values were calculated using two-tailed Student's *t*-test, moderated by Benjamini–Hochberg's method. FDR = 0.1, s0 = 2. The full lists are shown in Dataset S1B.

Scale bars: **(A)** to **(C)**: 0.5 μm.

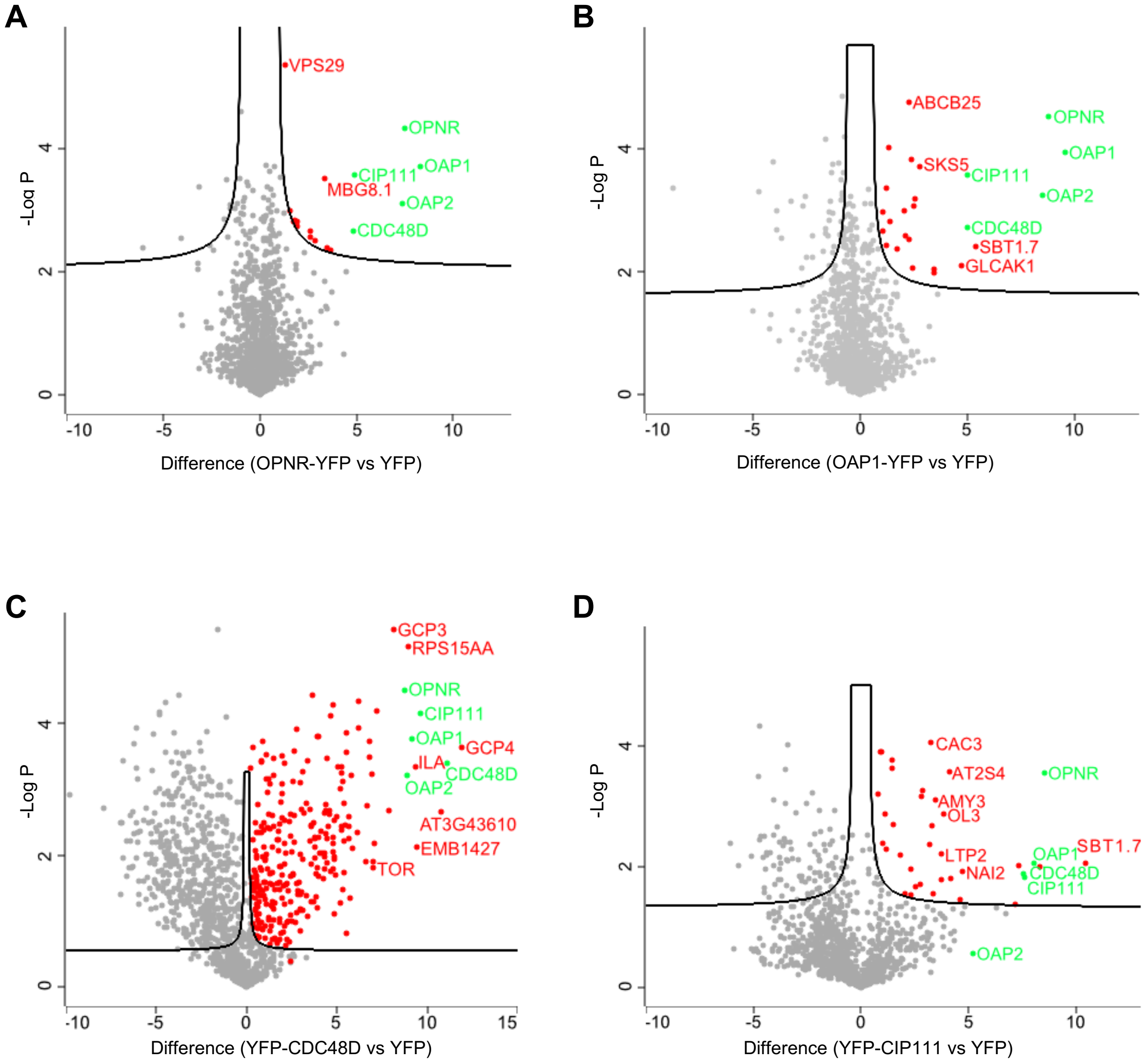

Supplementary Fig. 5. Proteomic analyses of OPNR-YFP, OAP1-YFP, YFP-CDC48D, and YFP-CIP111 interacting proteins.
GFP-Trap co-IP and mass spectrometry results with OPNR-YFP, OAP1-YFP, YFP-CDC48D and YFP-CIP111 serving as baits. Volcano plots for OPNR-YFP vs YFP **(A)**, OAP1-YFP vs YFP **(B)**, YFP-CDC48D vs YFP **(C)**, and YFP-CIP111 vs YFP **(D)**, with the -log10*P*-value shown on the y-axis and log2 intensity differences between the comparisons shown on the x-axis. Each dot represents a protein. Red and green dots represent significantly enriched proteins. Components of the OPNR complex are shown in green. *P*-values were calculated using two-tailed Student's *t*-test, moderated by Benjamini–Hochberg's method. FDR = 0.1, s0 = 0.1. The full lists are shown in Dataset S1C.

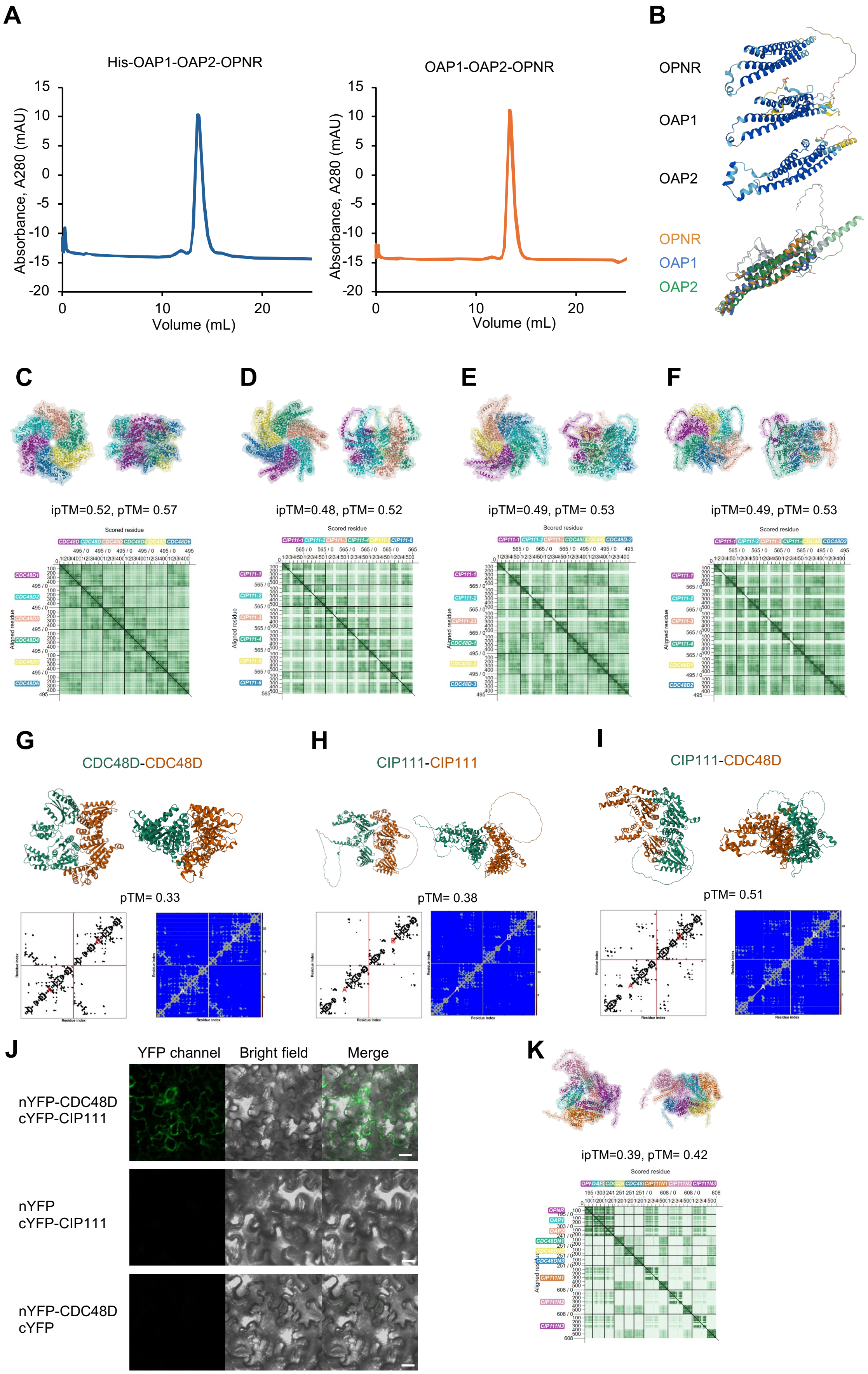

A
His-OAP1-OAP2-OPNR
OAP1-OAP2-OPNR
Absorbance, A280 (mAU)
Volume (mL)
B
OPNR
OAP1
OAP2
OPNR
OAP1
OAP2
C
D
E
F
ipTM=0.52, pTM= 0.57
ipTM=0.48, pTM= 0.52
ipTM=0.49, pTM= 0.53
ipTM=0.49, pTM= 0.53
G
CDC48D-CDC48D
pTM= 0.33
H
CIP111-CIP111
pTM= 0.38
I
CIP111-CDC48D
pTM= 0.51
J
YFP channel
Bright field
Merge
nYFP-CDC48D
cYFP-CIP111
nYFP
cYFP-CIP111
nYFP-CDC48D
cYFP
K
ipTM=0.39, pTM= 0.42

Supplementary Fig. 6. Predicted three dimensional structures of OPNR, OAP1, OAP2, CDC48D and CIP111.
(A) Size Exclusion Chromatography (SEC, Superdex 200 10/300 GL) analysis of His-OAP1-OAP2-OPNR and OAP1-OAP2-OPNR recombinant proteins showed a major peak at 13.4 mL and 13.57 mL, respectively. According to the calibration curve of the standard proteins Superdex 200 10/300 GL column, the size of the protein complexes are around 80 kDa.
(B) The AlphaFold predicted 3-D structures of OPNR, OAP1, and OAP2, including the alignment of the structures.
(C) to **(G)**: The AlphaFold predicted structures of the ATPase domains (D1 and D2) of 6 CDC48D **(C)**, 6 CIP111 **(D)**, 3 CDC48D and 3 CIP111 **(E)**, and 2 CDC48D and 4 CIP111 **(F)**. In each figure, the 3-D structure is shown from two different angles. The pTM and ipTM scores are also specified for each prediction. The predicted aligned error (PAE) provided for each figure how high confidence (dark green) and low confidence (pale green) regions. The N-terminus domain of CIP111 (N) and the two ATPase domains (D1 and D2) of CIP111 and CDC48D are labelled in **(C)**.
(G) to **(I)**: DMFold predicted structures of the CDC48D-CDC48D ATPases homodimer **(G)**, CIP111-CIP111 ATPase homodimer **(H)**, and CDC48D-CIP111 ATPases heterodimer **(I)**. The pTM scores, contact map (left) and distance map (right) are provided for each prediction.
(J) Bimolecular fluorescence complementation (BiFC) assay between CDC48D and CIP111. nYFP-CDC48D and cYFP-CIP111 were transiently co-expressed or were co-expressed with the free cYFP or nYFP in *Nicotiana Benthamiana* leaves. YFP fluorescence was detected by confocal laser scanning microscopy when nYFP-CDC48D was co-expressed with cYFP-CIP111 at three days after infiltration. Scale bars: 20 µm.
(K) The AlphaFold predicted structures of one OPNR-OAP1-OAP2 trimer, three N-termini and D1 domains of CIP111 and three N-termini and D1 domains of CDC48D.

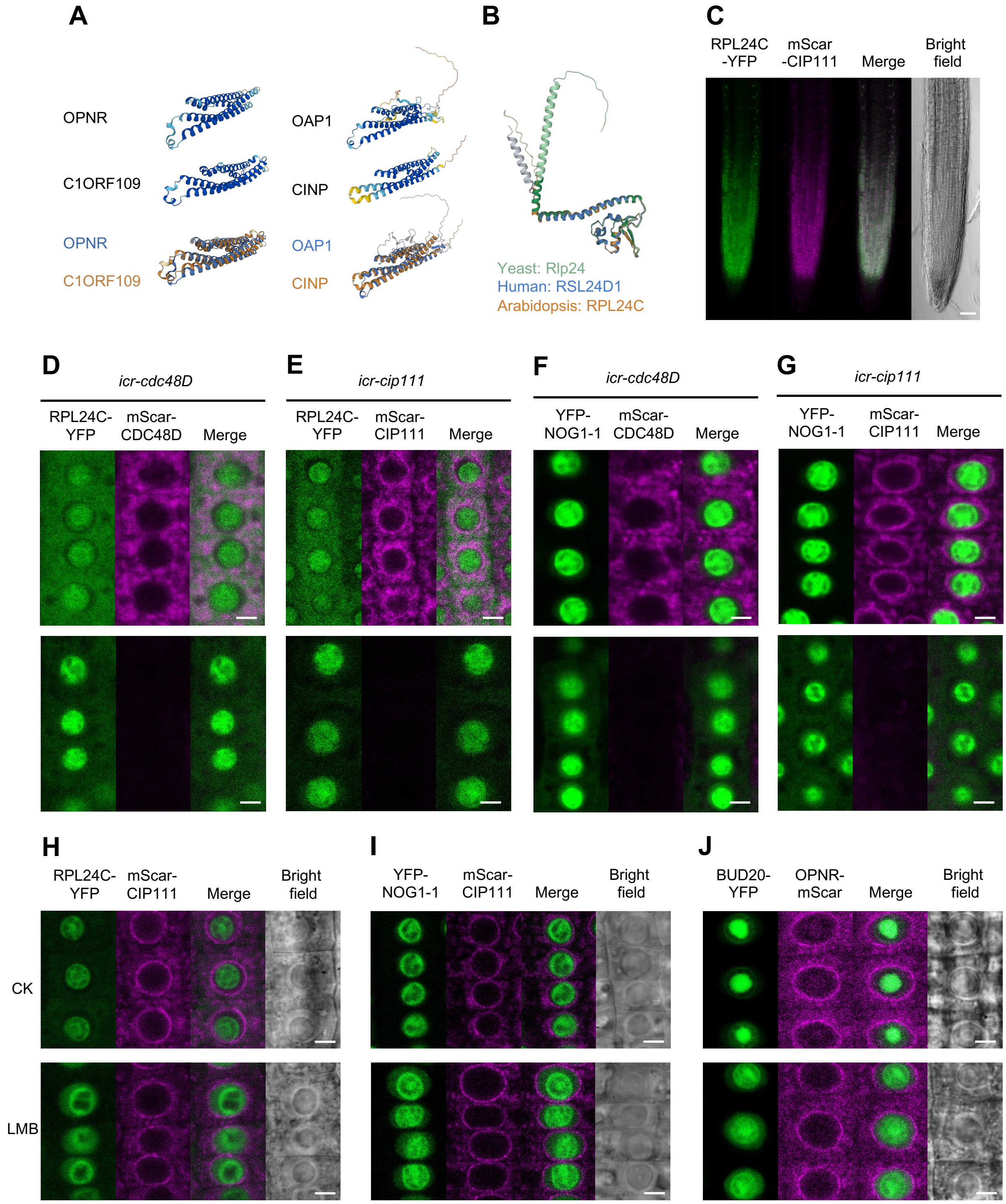
A
OPNR
C1ORF109
OAP1
CINP
OPNR
C1ORF109
OAP1
CINP
B
Yeast: Rlp24
Human: RSL24D1
Arabidopsis: RPL24C
C
RPL24C -YFP
mScar -CIP111
Merge
Bright field
D
icr-cdc48D
RPL24C-YFP
mScar-CDC48D
Merge
E
icr-cip111
RPL24C-YFP
mScar-CIP111
Merge
F
icr-cdc48D
YFP-NOG1-1
mScar-CDC48D
Merge
G
icr-cip111
YFP-NOG1-1
mScar-CIP111
Merge
H
RPL24C-YFP
mScar-CIP111
Merge
Bright field
CK
LMB
I
YFP-NOG1-1
mScar-CIP111
Merge
Bright field
J
BUD20-YFP
OPNR-mScar
Merge
Bright field

K

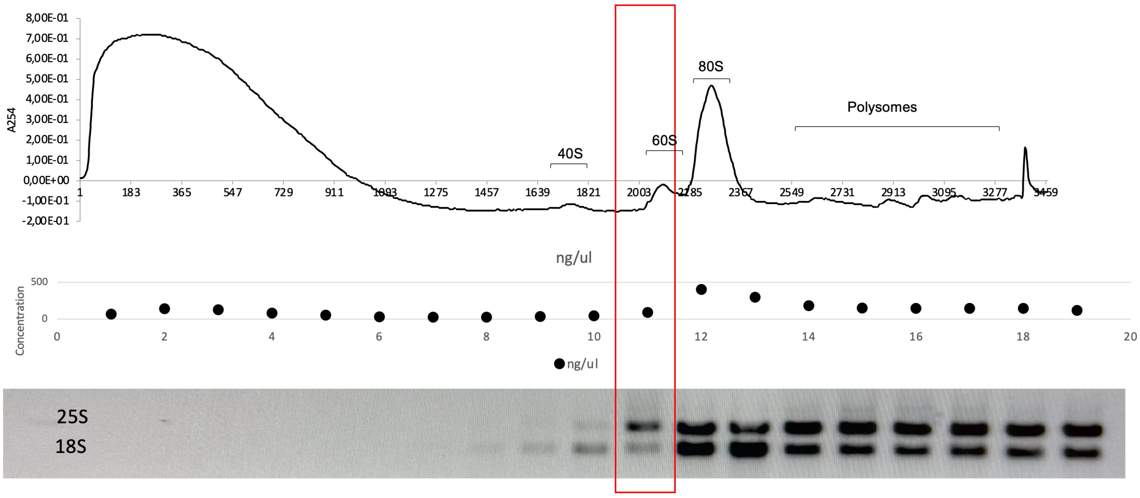

Supplementary Fig. 7. Support for RPL24C as the substrate of OPNR complex.

(A) The AlphaFold predicted 3-D structures of OPNR, C1ORF109, OAP1, and CINP. The alignments of OPNR-C1ORF109, and OAP1-CINP are also shown.

(B) The alignment of predicted structures of Arabidopsis RPL24C, human RSL24D1, and yeast *Saccharomyces cerevisiae* Rlp24.

(C) CSLM images of roots from seedlings co-expressing *pRPL24C:RPL24C-YFP* and *pCIP111:mScarlet-CIP111m*.

(D) and **(E)** CSLM images showing the localization of RPL24C-YFP before (upper panel) and after (lower panel) CRISPR/Cas9 induced mutation of *CDC48D* (*icr-cdc48D*) **(D)** and *CIP111* (*icr-cip111*) **(E)**.

(F) and **(G)** CSLM images showed the localization of YFP-NOG1-1 before (upper panel) and after (lower panel) CRISPR/Cas9 induced mutation of *CDC48D* (*icr-cdc48D*) **(F)** and *CIP111* (*icr-cip111*) **(G)**.

(H) to **(J)** localization of RPL24C-YFP **(H)**, YFP-NOG1-1 **(I)** and BUD20-YFP **(J)** before (upper panel) and after (lower panel) treatment of Leptomycin B (LMB).

(K) Ribosome profile and RNA gel example used to identify and isolate the 60S ribosome fraction.

Scar bars: **(C)**, 50 µm; **(D)** to **(J)**, 5 µm.

Supplemental Table 1. Primers and sgRNAs used in this study.

Applications	Primer names	Sequences (from 5' to 3')
**Primers for genotyping**		
opnr-1 T-DNA insertion	opnr-1-LP	ACTGGAGTCTCTATTGCGTTCC
	LB1.3	ATTTTGCCGATTTCGGAAC
opnr-1 T-DNA homolog	opnr-1-LP	ACTGGAGTCTCTATTGCGTTCC
	opnr-1-RP	CTTCAAGGTTGACGCTGAGAC
oap1 T-DNA insertion	oap1-LP	ACTTCGCGTTTGAACATCTCC
	LB1.3	ATTTTGCCGATTTCGGAAC
oap1 T-DNA homolog	oap1-LP	ACTTCGCGTTTGAACATCTCC
	oap1-RP	ATGAGCATCTCGCAGTTTCCG
oap2 CRISPR line genotyping	oap2-Fw	ATGGAAACGAGAATAGAACTG
	oap2-Rv	AAGTCCTGATAGAGATGTATGC
cdc48D CRISPR line genotyping	cdc48D-Fw	ATGTTGGAAACCGAAAGCAG
	cdc48D-Rv	GCAATACGAACATCTTGTTCTC
cip111 CRISPR line genotyping	cip111-Fw	ACCGCCTGCATCTCCAAGAA
	cip111-Rv	CCTGAAGGGAGTCAACTCTAAACA
**sgRNAs**		
OAP2	OAP1-sg1	TGGATCGTCGCTTGTTCTGC
	OAP1-sg2	TGTTACACGAAGCTGAGTAC
OAP2	OAP2-sg1	GCGGCTTAAATCATATATCG
	OAP2-sg2	TCACGCAGCTTGAAGAACTA
CDC48D	CDC48D-sg1	TCATCGACGACGAGACGGTT
	CDC48D-sg2	CAACAGTATCATCAACAGGC
CIP111	CIP111-sg1	GTTGCATCAGAATCTATCAG
	CIP111-sg2	TGCAGGCAAGCAGCGATAGA
	CIP111-sg3	CTAAGTCTAATGTATGATGG
	CIP111-sg4	AAGCGAGAAAGCTCTAGATG
OPNR	OPNR-sg1	GGAGGCACTTCAAGTTCGAT
	OPNR-sg2	GATGTCAGGGAGGAATTCTG

**Supplemental Table 1.** Primers and sgRNAs used in this study (continued).		
Applications	Primer names	Sequences (from 5' to 3')
**Primers for cloning**		
OAP1 Giboson cloning	pOAP1-greenF	TCATAACGTGACTCCCTTAACAGGAATAGAGAGGTTCATTTCAC
	OAP1-HRV-R	CCTTGGAAGAGTACTTCCAGGAAGGTGACTCTAATTTCTTCCCC
OAP1 GreenGate cloning	pOAP1-GGA-F	tagaagtgaagcttggtctcaacctCAGGAATAGAGAGGTTCATTTCAC
	pOAP1-GGA-R	tagggcgagaattcggtctcatgttcgaatacaaaacgagttttttgag
	OAP1-GGC-F	gaagtgaagcttggtctcaggctccATGGAAACGAGAATAGAAC
	OAP1-GGC-R	tagggcgagaattcggtctcactgaGTATATCCATTTCCATGCTC
OAP2 Giboson cloning	pOAP2-greenF	TCATAACGTGACTCCCTTAACTATGGAAGTCCTCGTGCCGT
	OAP2-R	TCAAAAGTGCCATCCATTTGAT
CDC48D Giboson cloning	pCDC48D-greenF	TCATAACGTGACTCCCTTAAAACTTCACTTCATAGCATTTAT
	pCDC48D-mScarR	GCCTCGCCCTTGCTCACCATACTAGTGAAGAACCAAACTTTGGG GA
	CDC48D-F	CTGGAAGTACTCTTCCAAGGACCAATGTTGGAAACCGAAAGCAG TGTCTG
	CDC48D-greenR	AACCGATGATACGAACGAAAGCTATGTAGCAGAAGCTACTAGTA ATTCAT
CDC48D GreenGate cloning	pCDC48D-GGA-F	tagaagtgaagcttggtctcaacctAACTTCACTTCATAGCATTTAT
	pCDC48D-GGA-R	tagggcgagaattcggtctcatgttGAAGAACCAAACTTTGGGGAAC
	CDC48D-GGC-F	gaagtgaagcttggtctcaggctccATGTTGGAAACCGAAAGCAGTG
	CDC48D-GGC-R	atgcgatcgcactagcggcacctgaTGTAGCAGAAGCTACTAGTAATTC
CIP111 Giboson cloning	pCIP111-greenF	TCATAACGTGACTCCCTTAAGGCTGATCCAATCATCTTGGC
	pCIP111-mScarR	GCCTCGCCCTTGCTCACCATACTAGTCTTTAGCTTTTTGTTAATC T
	CIP111-F	GCATGGACGAGCTGTACAAGGGATCCATGCCTTCGAAGAAGAA GCA
	CIP111-greenR	CGATGATACGAACGAAAGCTACGAAGCAATGTGGCGGCGAAG
CIP111 sgRNA targeting sites mutation	CIP111m-AR	cCTtATgctcTCactaGCtACATCGGCTACTCTTGCGATG
	CIP111m-BF	aGCtagtGAgagcATaAGgGGCTCTAAGATTTGGCTATCG
	CIP111m-BR	aCAaGCcAatAaactcAagTGaTTAACATCATCATAGCG
	CIP111m-CF	ctTgagttTatTgGCtTGtAAAGAGTTGTGTTTAGAGTTG
CIP111 GreenGate cloning	pCIP111-GGA-F	tagggcgagaattcggtctcatgttctttagctttttgttaatctacactc
	pCIP111-GGA-R	tagaagtgaagcttggtctcaacctGGCTGATCCAATCATCTTGGC
	CIP111-GGC-F	gaagtgaagcttggtctcaggctccATGCCTTCGAAGAAGAAGCAGTC
	CIP111-GGC-R	atgcgatcgcactagcggcacctgaCGAAGCAATGTGGCGGCG
pOPNR:OPNR-mScarlet Giboson cloning	pOPNR-greenF	TCATAACGTGACTCCCTTAATGTTCTACTGCTAAaaatcc
	OPNR-mScarletR	actgcctcgcccttgctcacTGGTCCTTGGAAGAGTACTT

Supplemental Table 1. Primers and sgRNAs used in this study (continued).

Applications	Primer names	Sequences (from 5' to 3')
**Primers for cloning**		
pGreen 0029 for Gibson cloning	pGreen-F	GGTGCCTAATGAGTGAGCTAACT
	pGreen-R	TTAAGGGAGTCACGTTATGACC
mScarlet for Giboson cloning	mScarlet-F	ATGGTGAGCAAGGGCGAGGCAGT
	HRV-mScarlet-F	ACTGCCTCGCCCTTGCTCACTGGTCCTTGGAAGAGTACTT
	OPNR-mScarlet-F	AAGTACTCTTCCAAGGACCAgtgagcaagggcgaggcagt
	mScarlet-R	CTTGTACAGCTCGTCCATGC
	mScarlet-trbcSR	GATGATACGAACGAAAGCTACTTGTACAGCTCGTCCATGC
	mScarlet-HRVR	CCTTGGAAGAGTACTTCCAGCTTGTACAGCTCGTCCATGC
rbcS terminator for Giboson cloning	TrbcS-F	TAGCTTTCGTTCGTATCATCG
	TrbcS-R	TAGCTCACTCATTAGGCACCGATTGATGCATGTTGTCAATC
