# Supplementary material for: Ribosome biogenesis in plants requires the nuclear envelope and mitochondria localized OPENER complex
Source: Nat Commun. 2025 Aug 7;16:7301. doi: 10.1038/s41467-025-62652-7 (PMC12332008; doi:10.1038/s41467-025-62652-7)
Supplement: Supplementary file 2 — Description of additional supplementary files [file 41467_2025_62652_MOESM2_ESM.pdf]

Description of Additional Supplementary Files

Title: Supplementary Data 1

Description: GFP-Trap co-IP and mass spectrometry results show log2 intensity differences between the comparisons between TurboID-YFP-CDC48D and TurboID-YFP. The -log10 P-values show the significands of the difference. The information of the candidate interactors was shown.

Title: Supplementary Data 2

Description: TurboID results show the –log10 P-value, the log2 inputated and normalized intensity, the log2 original intensity, and the log2 intensity differences of TurboID-YFP-CDC48D (CDC48D) and TurboID-YFP (CK). The intensity of each protein was normalized to the total signal of each sample. And the information of each protein was also shown.

Title: Supplementary Data 3

Description: GFP-Trap co-IP and mass spectrometry results show log2 intensity differences between the comparisons between OPNR-YFP, OAP1-YFP, YFP-CDC48D, YFP-CIP111 and YFP control. The -log10 P-values show the significands of the difference. The information of the candidate interactors was shown.

Title: Supplementary Data 4 and 5

Description: Results of 3D structure compare of OPNR (D) and OAP2 (E) with the AlphaFold predicted structure of human proteins in DALI. The Z scores and protein information were shown.

Title: Supplementary Data 6

Description: Proteomics data shows the log2 and normalized intensity, the log2 original intensity and the log2 intensity differences of 60S ribosome fraction of *icr-oap1* and *icr-cdc48D* vs Col-0.
